# Supplementary material for: Structural Properties of Epoxy–Silica Barrier Coatings for Corrosion Protection of Reinforcing Steel
Source: Polymers (Basel). 2022 Aug 25;14(17):3474. doi: 10.3390/polym14173474 (PMC9460649; doi:10.3390/polym14173474)
Supplement: Supplementary file 1 [file polymers-14-03474-s001.zip › polymers-1860480-supplementary.pdf]

polymers

MDPI

Supplementary File

Structural Properties of Epoxy-Silica Barrier Coatings for Corrosion Protection of Reinforcing Steel

Mayara Carla Uvida, Adriana de Araújo Almeida, Sandra Helena Pulcinelli, Celso Valentim Santilli and Peter Hammer *

São Paulo State University (UNESP), Institute of Chemistry, 14800-060, Araraquara/SP, Brazil; mayara.uvida@unesp.br, adriana.a.almeida@unesp.br, sandra.h.pulcinelli@unesp.br, cv.santilli@unesp.br

* Correspondence: peter.hammer@unesp.br; Tel.: +55 16 3301 9887

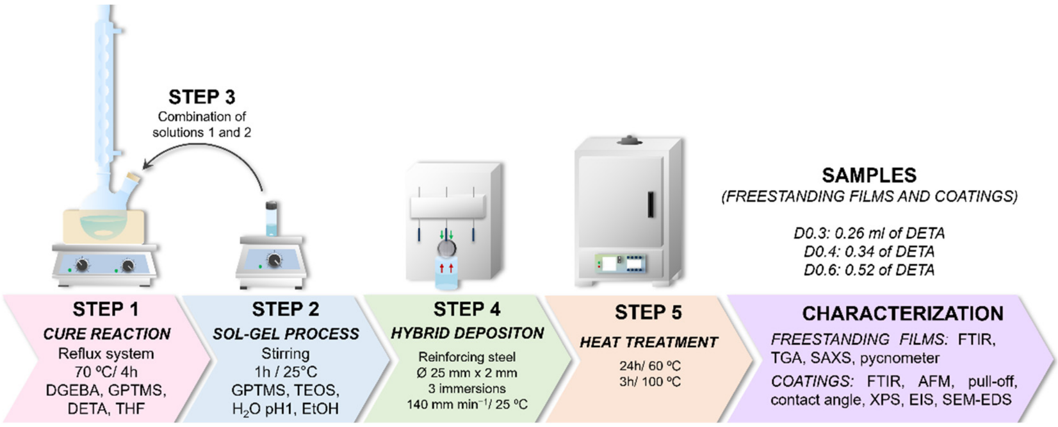

Figure S1. Experimental procedure for epoxy-silica hybrids.

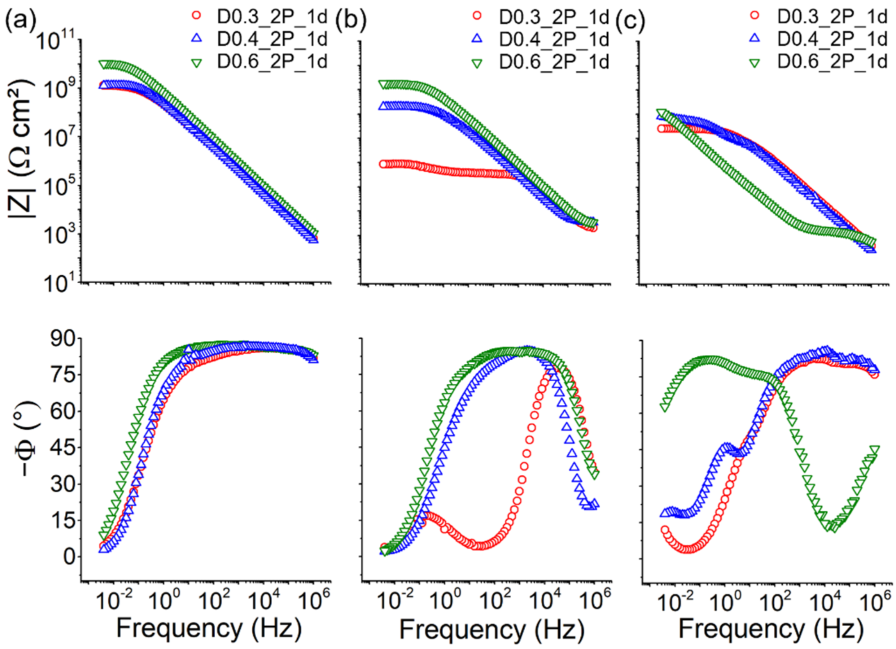

Figure S2. Bode plots for duplicates of epoxy-silica hybrid coatings prepared with different DETA/DGEBA ratios after 1 day of exposure in solution: (**a**) 3.5 wt% NaCl, (**b**) SCPS1 (pH 8) and (**c**) SCPS2 (pH 14).

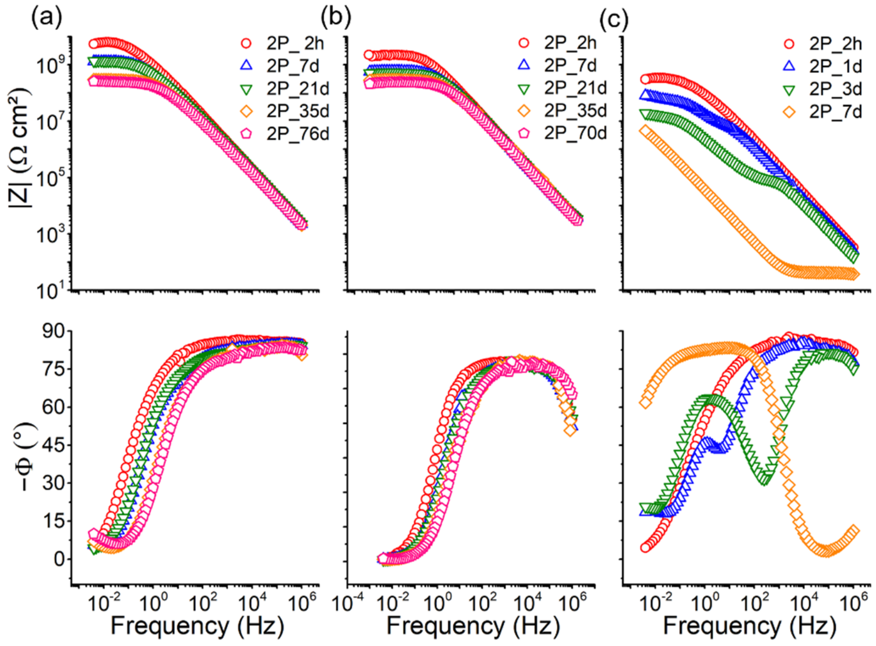

Figure S3. Bode plots for duplicates of epoxy–silica hybrid coating prepared with DETA/DGEBA = 0.4 as a function of immersion time in: (**a**) 3.5 wt % NaCl, (**b**) SCPS1 (pH 8), and (**c**) SCPS2 (pH 14) solution.
